# A Mendelian randomization study on the causal effects of circulating cytokines on the risk of vitiligo

**DOI:** 10.3389/fmed.2024.1375339

**Published:** 2024-04-17

**Authors:** Chengling Liu, Xingchen Liu, Haiming Xin, Xin Li

**Affiliations:** ^1^Center of Burns and Plastic Surgery and Dermatology, The 924th Hospital of Joint Logistics Support Force of the PLA, Guilin, China; ^2^Department of Pathology, Changhai Hospital, Naval Medical University, Shanghai, China

**Keywords:** vitiligo, cytokine, Mendelian randomization, IL-4, genome-wide association study

## Abstract

**Background:**

Accumulating evidence reveals an association between circulating cytokine levels and vitiligo. However, the causal association between circulating cytokine levels and vitiligo remains unrevealed.

**Methods:**

We performed a two-sample Mendelian randomization (MR) analysis using a genome-wide association study of the 41 cytokines dataset, which was conducted with 3 Finnish cohorts (*n* = 8,293). Vitiligo data were acquired from strictly defined vitiligo data collected by FinnGenbiobank analysis, which included 207,613 European ancestors (131 vitiligo patients, 207,482 controls). The inverse-variance weighted (IVW) method, weighted median (WME), simple model, weighted model, and MR-Egger were used to determine the changes in vitiligo pathogenic cytokine taxa, followed by sensitivity analysis, including horizontal pleiotropy analysis. The MR Steiger test evaluated the strength of a causal association, and the leave-one-out method was used to assess the reliability of the results. The possibility of reverse causality was also investigated using a reverse MR study.

**Results:**

We observed that rising IL-4 levels generated an enhanced probability of vitiligo in IVW (OR 2.72, 95%CI 1.19–6.22, *p* = 0.018). According to the results of the MR analysis, there were causal links between IL-4 and vitiligo. Results were steady after sensitivity and heterogeneity analyses.

**Conclusion:**

Our research reveals that a genetically determined increased level of circulating IL-4 may be linked to a higher risk of developing vitiligo. The development of innovative treatment approaches (such as tofacitinib or dupilumab) that focus on blocking IL-4 as a novel way of preventing and treating vitiligo is significantly impacted by our findings.

## 1 Introduction

Vitiligo, which targets melanocytes that produce pigment, causes depigmented areas that appear as white spots (1). There is no sex bias in vitiligo, a common dermatological condition with an incidence rate of 0.1 to 2% worldwide (2). There are several therapeutic options available, but difficulties still exist since not all patients react to the medications that are offered, relapses are frequent, and complete repigmentation is seldom achieved (3).

Vitiligo, a multifactorial condition, involves autoimmune reactions, inflammatory mediator production from environmental stimuli, and hereditary predisposition (4, 5). Although there is no widely known origin of vitiligo, it is characterized by the death of melanin and melanocytes in the skin, causing depigmented, asymptomatic macules with well-defined borders (6). Meanwhile, autoreactive CD8 + T lymphocytes drawn to proinflammatory cytokines are thought to facilitate melanocyte death in vitiligo by killing the pigment-producing cells of melanocytes via interferon-gamma (IFN-γ) signaling (7). According to recent studies, the maintenance of vitiligo is accompanied by a significant change in the expression of inflammatory cytokines in afflicted skin compared to unaffected skin. A novel idea postulates that the pathogenesis of autoimmune ailments, specifically the pathogenesis of vitiligo, may be caused by the twisting of responses to Th1 or Th17 and disengaging from Tregs and Th2 cells (8). The first sign that cellular immunity played a role in the etiology of vitiligo was the finding of a T-cell infiltration in the lesion margins in inflammatory vitiligo (9). According to Yu et al., interleukin-6 (IL-6) may be a critical factor in melanocytic cytotoxicity since vitiligo patients produced significantly more IL-6 and IL-8 while releasing less TNF-α and IFN-γ (10). Among the cytokines, IL-4 is a crucial cytokine that aids in the humoral immune response, the differentiation of naive Th cells into Th2, the autocrine proliferation of differentiated Th2 cells, and the maintenance of Th2 lymphocytes (11). The function of IL-4 in the vitiligo progression is still ambiguous, nevertheless. Levels of IL-4 can be both elevated (11–13) and lowered (14) throughout the pathogenesis of vitiligo, according to a significant number of studies. Does the illness’s categorization, stage, or degree of activity influence levels of IL-4? Understanding how IL-4 behaves and works differently during the vitiligo process is crucial to effectively deploying IL-4 antagonists as a novel therapeutic approach.

Mendelian randomization (MR) evaluates the causality of a particular exposure on an outcome of interest by using genetic variation that is closely related to a potential exposure as instrumental variables (IVs) (15). Unlike observational research, MR is unaffected by reverse causality or confounding variables. Tens of thousands of genetic variations that are strongly related to complex features have been discovered in the last 10 years by genome-wide association studies (GWASs) (16). These discoveries make the genetic basis of complex features apparent. Hence, Mendelian investigation is a causal inference technique with robust supporting data for determining the causal link between cytokines and vitiligo. In this study, we used a two-sample MR method to analyze aggregated information on inflammatory cytokines and vitiligo from genome-wide association research carried out in three large cohorts to determine the causal relationship between inflammatory cytokines and vitiligo risk.

## 2 Methods

### 2.1 Ethics statement and overall study design

**Figure 1:**
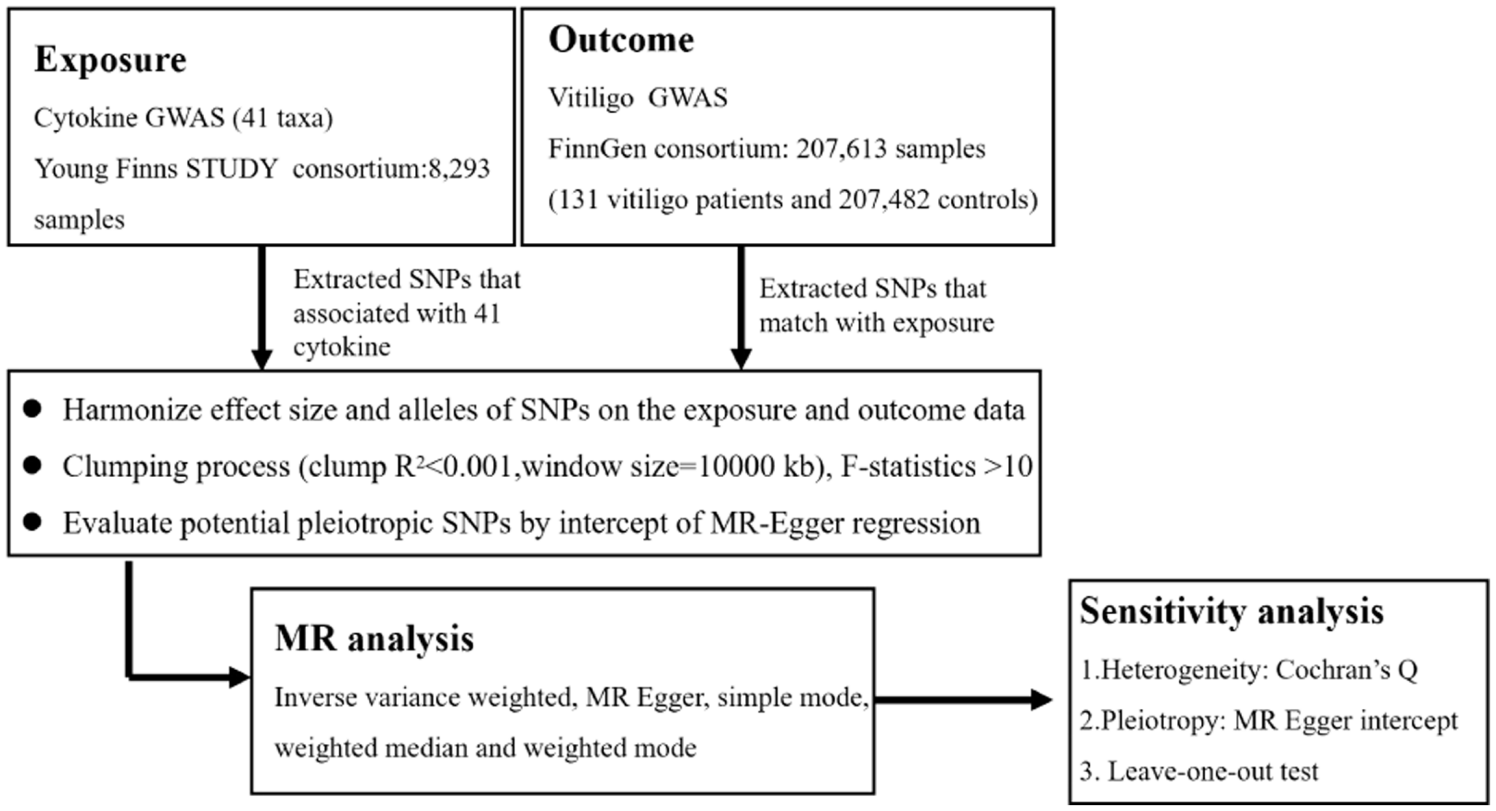
Flowchart of this Mendelian randomization study.

We used the GWAS’s published summary statistics for our analysis. No new information was acquired, and no new ethical analysis was conducted. The flow chart in Figure 1 depicts the entire procedure that we investigated. In simple terms, we conducted a two-sample MR investigation to assess the relationship between cytokines and vitiligo. Three fundamental presumptions serve as the foundation for MR’s validity (17): (1) relevance - the relationship between genetic variants and exposure was robust; (2) independence - the genetic variants were independent of confounding factors affecting exposure and outcome; and (3) exclusion restriction - the genetic variants influenced the risk of the outcome through exposure rather than other potential pathways. To determine any bidirectional causal links between the cytokines and vitiligo, we utilized a two-sample MR computational model (the random-effects inverse-variance weighted (IVW), MR-Egger regression, weighted median (WME), weighted model, and MR Steiger). The heterogeneity test, pleiotropy test, MR-PRESSO, reverse MR, and leave-one-out sensitivity analyses were carried out successively.

### 2.2 Data sources

The GWAS summary statistics used in the study are presented in Table 1. The individuals from the data sources were of European ancestry.

**Table 1:** Summary of genome-wide association studies (GWAS) datasets.

Trait	Year	Population	Sources	Sample sizes	Cases	Control
Exposure						
Cytokine	2021	European	Finnish cohorts	8,293	–	-
Outcome						
Vitiligo	2021	European	FinnGen	207,613	131	207,482

#### 2.2.1 Cytokines

We accessed genome-wide association summary-level data for vitiligo and 41 circulating cytokines. The Cardiovascular Risk in Young Finns study, FINRISK 1997, and FINRISK 2002 provided 10.7 million SNPs and 8,293 Finnish people who made up the summary-level data for 41 cytokines (18). The Bio-Rad Bio-Plex Pro Human Cytokine 27-plex Assay and 21-plex Assay, along with the Bio-Plex 200 reader and Bio-Plex 6.0 software, were used to measure the cytokine levels. Reference haplotypes from the first phase of the 1,000 Genomes Project were used to infer genotypes (19). After correcting for age, sex, and body mass index, a linear regression between cytokine levels and SNPs was used to determine the single-variant link.

#### 2.2.2 Vitiligo

Summary statistics for vitiligo in individuals of European ancestry were obtained from the publicly available GWAS analyses. The study investigated vitiligo cases (131) and controls (207,482), which included over 16 million genetic variants. Detailed information on the study has been described in the previous study (20). The study was conducted with the signed informed consent of every participant, and each regional institutional review board oversaw it.

### 2.3 Selection of genetic instrumental variables

We selected single-nucleotide polymorphisms (SNPs) associated with cytokines with a relatively significant level (*p* < 5 × 10^−8^) (20). Then, we applied chain disequilibrium r^2^ < 0.001 within a distance of 10,000 kb as a cutoff of linkage disequilibrium for respective independence before being used as primary genetic instruments. After coordinating with responsive results, every pair of combinations was retrieved for additional analysis. To detect bias from weak instrumental factors, the *F* statistic of IVs was determined (*F* = R^2^(n-k-1)/k(1-R^2^)) (*n* is the sample size, k is the number of included IVs, and *R*^2^ is the exposure variance explained by the selected SNPs). The *F* statistic >10 was seen as evidence of the absence of bias from subpar instrumental factors. Finally, a reverse causality study was performed to investigate the reverse causal relationship, and a nominal causal effect was defined as one with a *p*-value between 0.05 and the corrected value.

### 2.4 Statistical analysis

The principal analysis used the IVW technique to get an unbiased estimate of the causal link between cytokines and vitiligo. Most of the time, exponential odds ratios (ORs) and associated confidence intervals (CIs) were utilized to assess the effects of causation. Statistics were considered significant if the *p*-value was <0.05. To further quantify causal effects under various circumstances, the WME, MR-Egger, simple mode, and weighted mode approaches were also used. The WME approach may combine information on several genetic variants into a single causal estimate and give a reliable estimate if at least half of the weight is derived from reliable IVs (21). If genetic changes show directional pleiotropy, the MR-Egger method might measure the causative effect (22). The intercept of the MR-Egger regression and MR pleiotropy residual sum and outlier (MR-PRESSO) was calculated to assess horizontal pleiotropy. Pleiotropy is unlikely to affect the causal analysis, according to the *p*-value of >0.05. The Cochran’s Q-test, used to identify heterogeneity among IVs, was created using the IVW estimation approach. We also applied the MR pleiotropy residual sum technique to assess horizontal pleiotropy and exclude likely outliers. The leave-one-out technique, which systematically removed one of the SNPs and used the other SNPs as IVs for two-sample MR analysis, was used to determine the extent of a single SNP’s causal association impact (23). The MR Steiger directionality test was performed to fully evaluate the relationship between exposure and results. According to the MR Steiger method, an appropriate genetic variation should account for more variation during exposure than during the result. This method ensures that the genetic tools are proper for a valid MR investigation and facilitates the detection of any reciprocal effects (24). Finally, we carried out a reverse MR analysis to examine the effect of vitiligo on the identified cytokines. SNPs related to vitiligo were used as IVs.

We believed there to be a significant causal relationship between systemic cytokines and vitiligo if the following criteria were met: The five approaches produced consistent estimates, the IVW method showed a significant difference (*p* < 0.05), the Cochran’s Q-test, MR-Egger, and MR-PRESSO were not substantial (*p* > 0.05), and the MR Steiger directionality tests confirmed TRUE. All MR analyses were performed using the R software’s ‘TwoSampleMR’ package (version 4.2.2). The statistical power is inevitably decreased since there is a relative dearth of sufficient data in cytokines and vitiligo GWAS datasets. We hope that more data will be available for verification in the future.

## 3 Results

### 3.1 Selection of instrumental variables

SNPs of cytokines were used after screening at a low threshold (*p* < 5 × 10^−8^) and LD clumping. In Supplementary Table S1, the final SNPs for the cytokine trait were shown in detail. There was no mild instrument bias, as indicated by the fact that all of the F statistics for the IVs were more than 10.

### 3.2 Causal impact of cytokine on vitiligo

**Figure 2:**
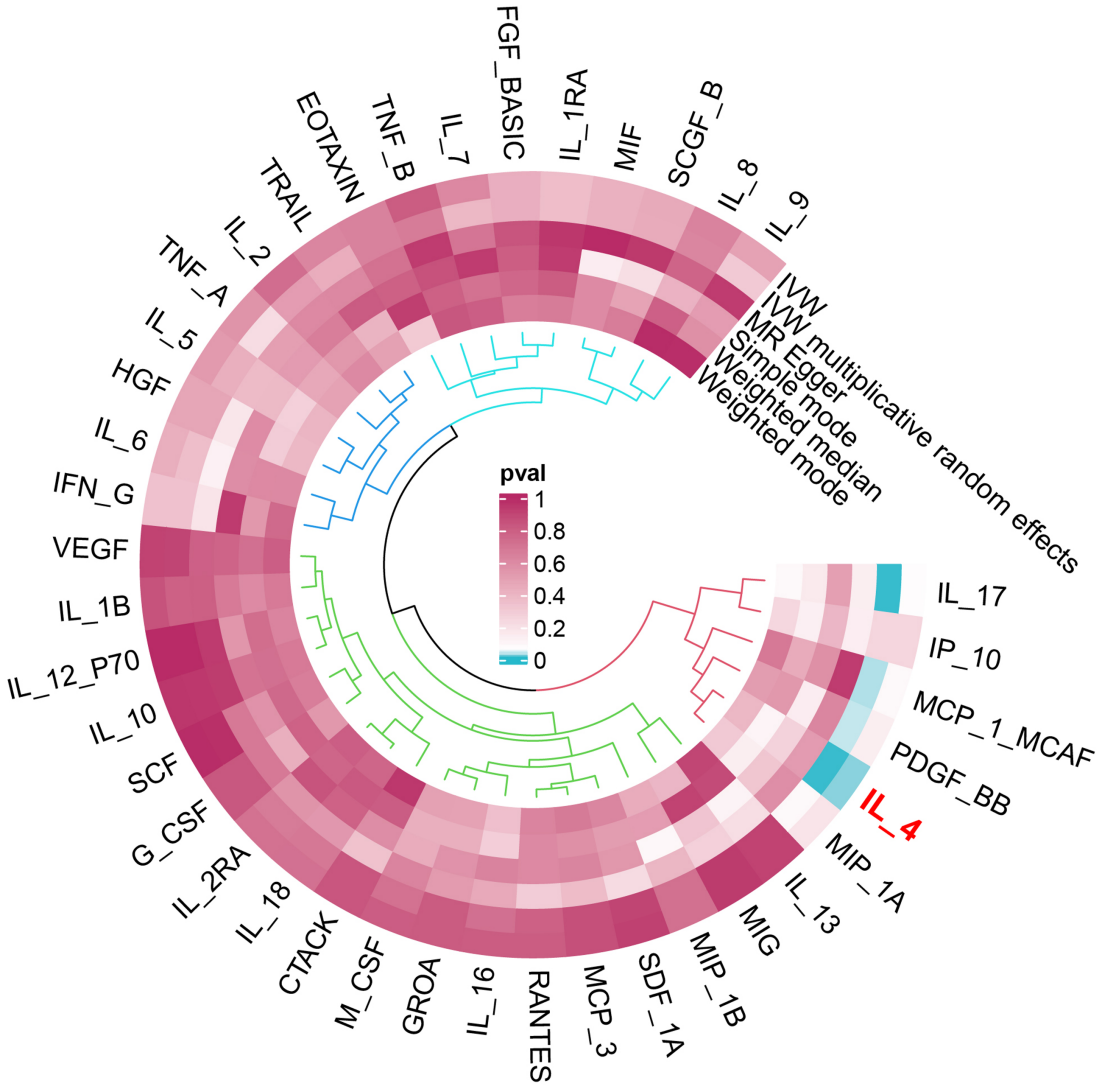
Overview of the causal role of cytokines in vitiligo. The cyan process color indicates statistical significance (*p* < 0.05). IVW, inverse-variance weighted method.

An overview of the causal effect of 41 cytokine taxa on vitiligo is shown in Figure 2. Of all the cytokines, only one cytokine was selected for further MR analyses. Furthermore, 14 independent SNPs were associated with IL-4. SNP detailed messages (SD, *R*^2^, and *F*) of significant genera in MR analyses are shown in Table 2.

**Table 2:** SNP message of significant cytokines.

Cytokine	SNP	SD	*R* ^2^	*F*
IL-4	rs17713451	0.578	0.045	384.528
	rs79597994	0.027	0.998	3809002.778
	rs73023729	0.172	0.522	8935.224
	rs10512267	0.443	0.033	282.789
	rs2073438	0.343	0.057	496.821
	rs6969391	0.425	0.032	266.315
	rs2849346	0.443	0.029	243.742
	rs9941733	0.371	0.089	794.965
	rs9508291	0.081	0.810	34844.444
	rs6765768	0.452	0.030	253.446
	rs117146485	0.099	0.892	67411.041
	rs12238729	0.066	0.985	229615.215
	rs116705532	0.072	0.977	341494.141
	rs13106889	0.199	0.262	2906.190
	rs17713451	0.262	0.317	3797.109
	rs79597994	0.578	0.045	384.528

SD, standard deviation; SNP, single-nucleotide polymorphism.

**Figure 3:**
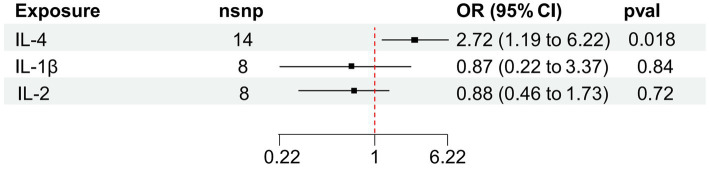
Forest plot of cytokines associated with vitiligo identified by the inverse-variance weighted method **(A,B)**. SNP, single-nucleotide polymorphism; OR, odds ratio; and CI, confidence interval.

We found that IL-4 (OR 2.72, 95%CI 1.19–6.22, *p* = 0.018) has an increased risk of developing vitiligo (Figures 3A,B). For all outcomes, the MR Steiger directionality tests revealed a similar pattern from cytokine to vitiligo (Table 3).

**Table 3:** Significant MR analysis results.

Bacterial taxa	MR method	No. SNP	OR	95%CI	*p*-value	P for MR-PRESSO global test
IL-4	IVW	14	2.72	1.44–5.12	0.00197	
	Weighted median		2.41	0.83–6.93	0.1025	
	MR-Egger		1.80	0.32–10.14	0.5165	
	Simple mode		3.46	0.44–27.16	0.5035	
	Weighted mode		2.40	0.35–16.40	0.3863	
	MR-PRESSO					0.465

OR, odds ratio; CI, confidence interval; SNP, single-nucleotide polymorphism; MR, Mendelian randomization; MR-PRESSO, MR pleiotropy residual sum and outlier.

### 3.3 Sensitivity analysis

**Figure 4:**
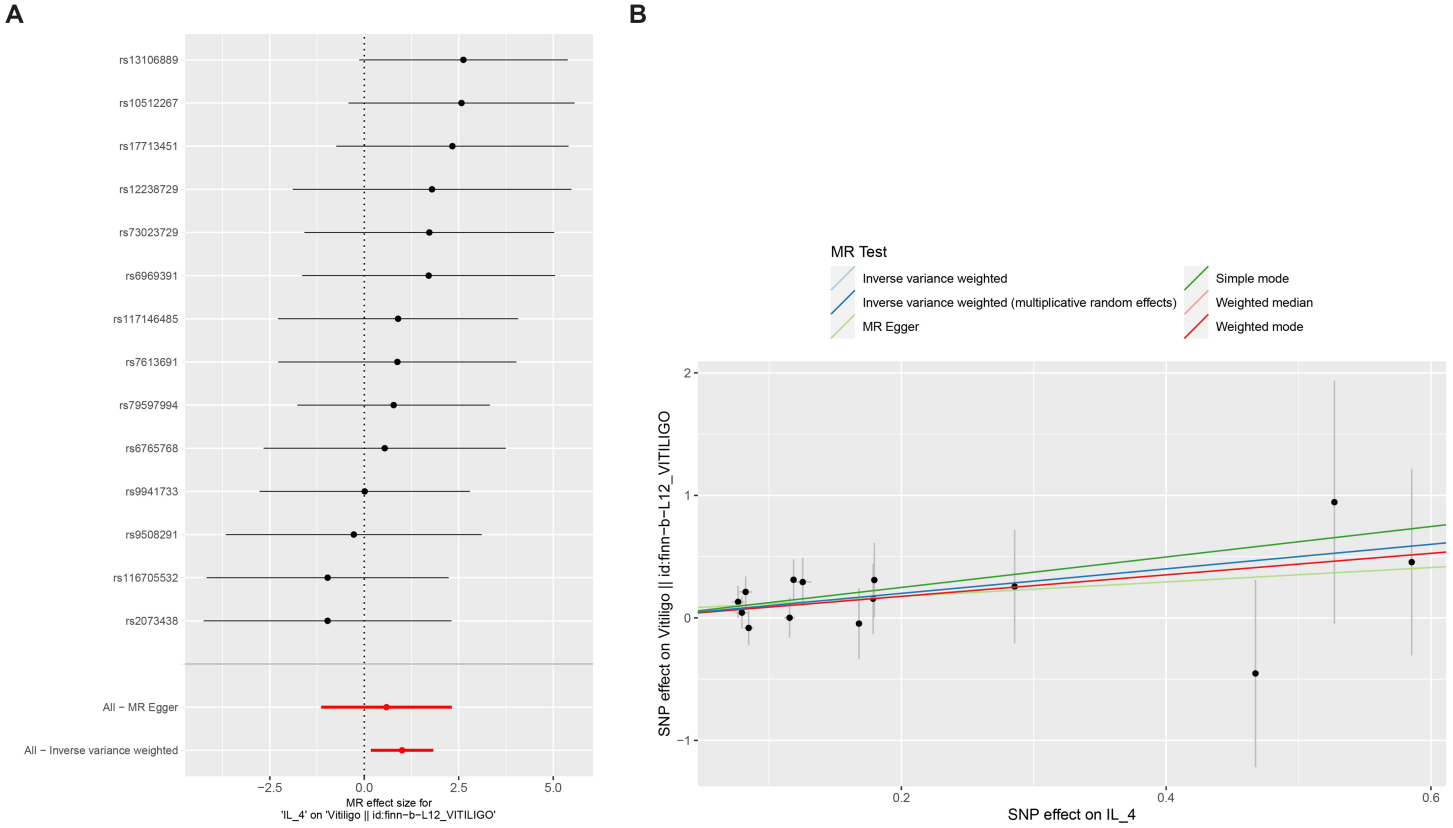
Scatter plots of cytokines associated with the risk of vitiligo.

**Figure 5:**
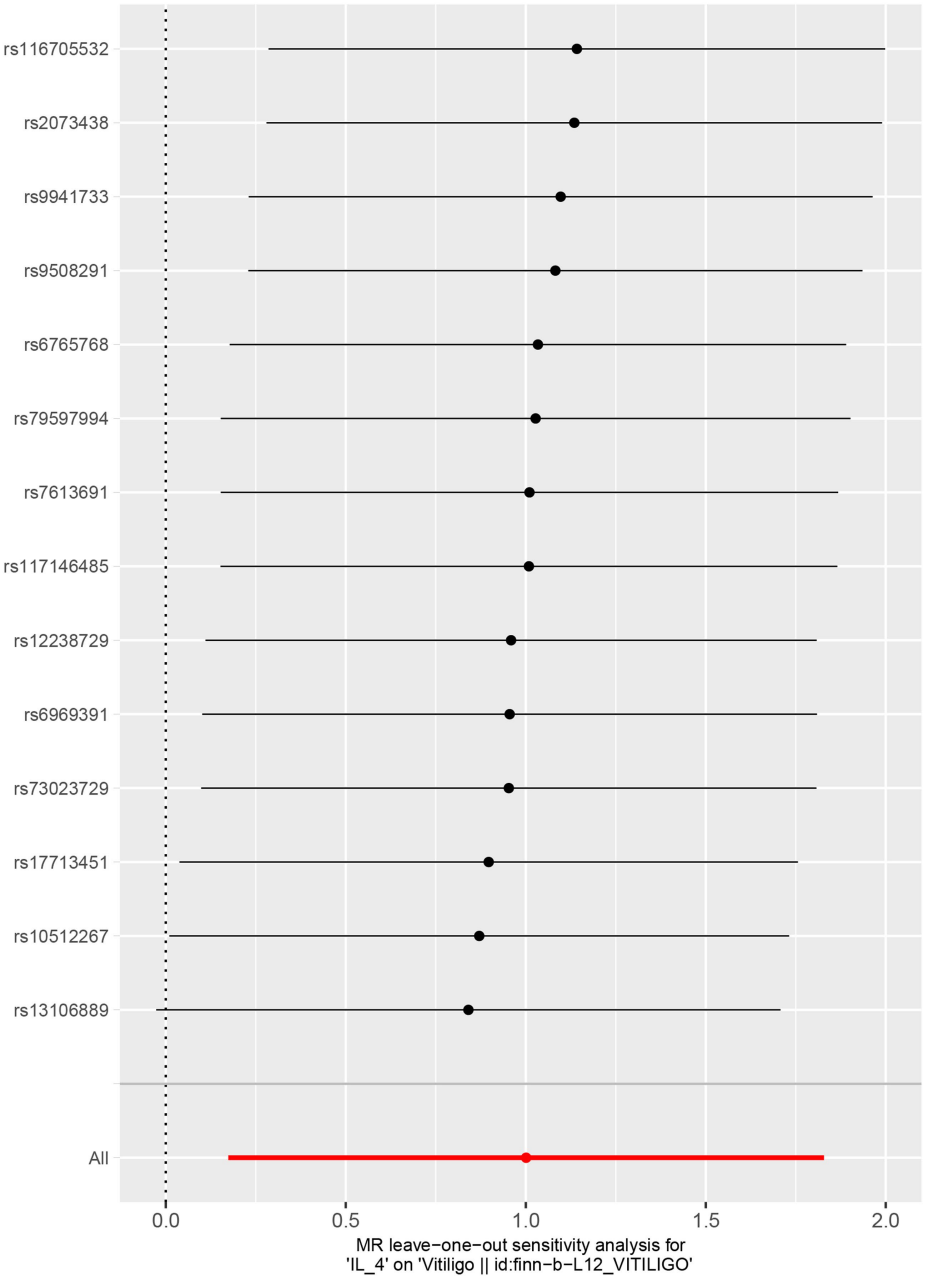
Leave-one-out analysis of cytokines associated with the risk of vitiligo.

**Figure 6:**
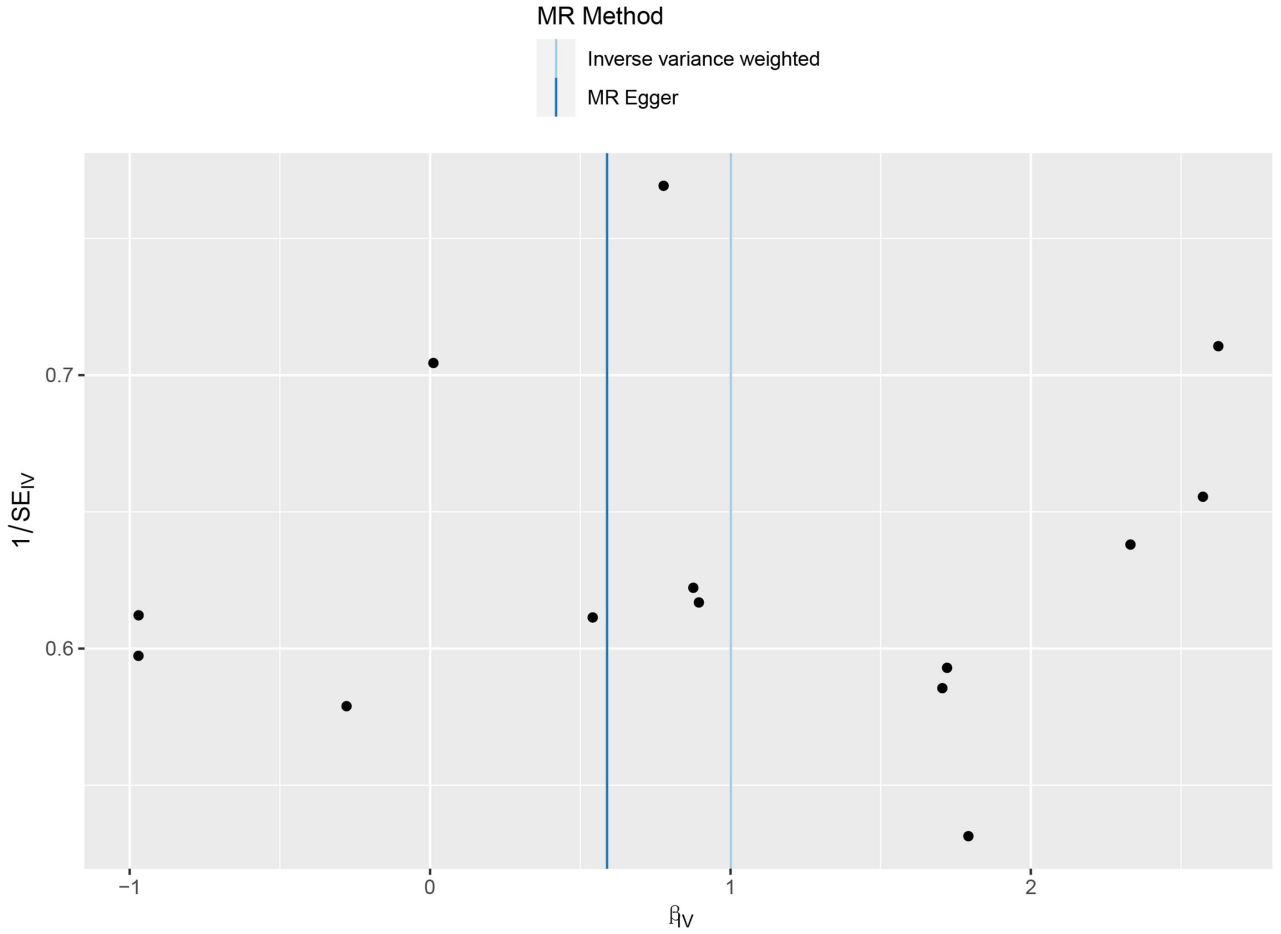
Funnel plots of cytokines associated with the risk of vitiligo.

No heterogeneity was found within the IVs of the cytokine by Cochrane’s Q-test (Table 4). The MR-Egger regression intercepts and MR-PRESSO indicated no horizontal pleiotropy or outlier values (*p* > 0.05). The scatter plots revealed that IL-4 may have a detrimental influence on vitiligo protection. The IVW method, MR-Egger, WME, weighted mode, and simple mode were the methods of MR analysis that were described in the scatter plots. Positive markers of the association between the cytokines and vitiligo were discovered to be the lines sloping upward from left to right. In contrast, protective cytokines were found to be those sliding downward from left to right (Figure 4). In the “leave-one-out” analysis of the cytokine IVs for vitiligo, there were no possible outliers (Figure 5), suggesting that the found causal link was not impacted by a single IV. Additionally, for whatever outcome, the funnel plots revealed no discernible horizontal pleiotropy (Figure 6). The reverse MR data analysis showed that vitiligo had no causal effect on IL-4 (Table 5).

**Table 4:** Sensitivity analysis of cytokine associated with vitiligo.

Exposure	SNPs	MR-Egger intercept		Cochrane’s Q IVW		Cochrane’s Q Egger		Correct causal direction
		Intercept value	*p*-value	*p*-value	*p*-value	*q*-value	*p*-value	
IL-4	14	−0.083	0.635	2.94	0.709	2.68	0.613	True

SNPs, single-nucleotide polymorphisms; IVW, inverse-variance weighted; MR, Mendelian randomization.

**Table 5:** Reverse causal association between vitiligo and cytokines.

Cytokines (outcome)	MR method	No. SNP	OR	95%CI	*p*-value	P for MR-PRESSO global test
IL-4	IVW	50	1.01	0.98–1.02	0.859	
	Weighted median		0.99	0.97–1.02	0.889	
	MR-Egger		1.00	0.98–1.02	0.822	
	Simple mode		0.83	0.37–1.82	0.639	
	Weighted mode		0.82	0.37–1.82	0.627	
	MR-PRESSO					0.477

SNPs, single-nucleotide polymorphisms; IVW, inverse-variance weighted; MR, Mendelian randomization; MR-PRESSO, MR pleiotropy residual sum and outlier.

## 4 Discussion

Studies have identified a strong correlation between circulating cytokines and vitiligo, although the causes of cytokines’ link with vitiligo are unidentified. In this study, we utilized large-scale GWAS for 41 circulating cytokines and vitiligo and assessed the causal association between circulating cytokines and vitiligo using two-sample MR techniques. We discovered an affinity between raised IL-4 levels and a higher chance of developing vitiligo (OR 2.72, 95%CI 1.19–6.22, *p* = 0.018). Pleiotropy analysis was used to find any significant pleiotropic variations among the selected cytokine instrumental variants in the datasets. Notably, the outcomes of five MR analysis methods reveal that the particular cytokine instrumental alterations greatly influence the risk of vitiligo without using other processes. These results imply that IL-4 and vitiligo are related causally.

Vitiligo is characterized as a mucocutaneous, immune-mediated, restricted, or disseminated illness and is characterized by the formation of hypochromic or achromic macules and a decline in quality of life (25). Numerous theories have been proposed to elucidate the pathophysiology of vitiligo. Molecular adhesion dysfunction, oxidative stress, autoimmune, auto-inflammatory, neurological, apoptotic, and multifunctional processes are only a few of these (26). However, most experts concur that vitiligo etiology and progression are significantly influenced by immune response and T-cell tolerance abnormalities (27). Furthermore, vitiligo is defined by the presence of an altered immunological balance, which is predominantly seen in an imbalance between the cytokines expressed by Treg/Th2 lymphocyte subsets (IL-4) and Th1/Th17 lymphocyte subsets (TNF-α, IFN-γ, IL-17, and IL-8) (28, 29). Early histopathology of vitiligo lesions indicated CD8+ T cell-dominant lymphocytic infiltrated around the margin of the depigmented lesions, where the disease was most active, in a prior analysis (30). The generation of the cytokine IFN-γ is crucial to developing the disorder in terms of the processes by which CD8 + T cells result in vitiligo. Additionally, a gene expression investigation of human lesional skin showed that IFN-γ-induced genes, including IFNG, were mostly upregulated (31, 32). The IFN-γ chemokine route is necessary for maintaining existing lesions and initiating and progressing vitiligo, making it a prospective treatment target that has shown positive results in clinical studies (33). On the other hand, studies have shown that a dysregulation of the Th1/Th2 balance caused by melanocytes secreting IFN-γ and IL-4 causes the demise of melanocytes (34, 35). Thus, the significance of Th2 cells and the cytokines IL-4 in the development of vitiligo sparks researchers’ attention.

The autoimmune depigment disorder is believed to be caused by a large-scale loss of melanocytes. One of the processes behind depigmentation in vitiligo is thought to be melanocyte death, which has been accelerated by IL-17 and other cytokines (36). According to a variety of studies, keratinocytes are a mediator of the detrimental impacts of oxidative stress. They release cytokines that attract autoreactive T lymphocytes and interfere with melanocyte signal transduction, which causes melanocyte death (37, 38). Choi et al. found that by suppressing the transcription and translation of genes related to melanogenesis, such as dopachrome tautomerase and microphthalmia-associated transcription factor (MITF), IL-4 directly reduced melanogenesis through the JAK2-STAT6 signaling pathway (39). Furthermore, Li′s study demonstrated that IL-33, a recently identified member of the IL-1 family, may function to promote melanocyte death in vitiligo skin with the increasing production of IL-4 (40). Thus, one of the possible mechanisms by which IL-4 plays a significant and vital role in vitiligo is the stimulation of the necrosis of melanocytes. According to Khan et al., vitiligo sufferers’ blood levels of IL-4 were higher than those of healthy controls (41). Imran et al. (12) discovered concurrently that vitiligo patients had considerably higher levels of IL-4 mRNA, serum IL-4, IgE, and the polymorphisms of the IL-4 gene may be genetic risk factors for susceptibility toward vitiligo. Grimes et al. found that IL-4 mRNA was expressed more in vitiligo patients than in healthy controls, although the difference did not achieve statistical significance (31). With the development of small molecule drugs, the Janus kinase/signal (JAK) transducer and activator of the transcription signaling pathway have been suggested as a promising therapeutic target in vitiligo (42). JAK1 and JAK3 were shown to be overexpressed in vitiligo patients’ skin compared to control skin in a stepwise manner that increased from control to non-lesional, perilesional, and lesional skin, according to Motaleb et al. Additionally, JAK3 had a significantly greater expression (43). The only JAK protein that can phosphorylate c receptor-carrying receptors is JAK3, and only receptors for IL-2, IL-4, IL-7, IL-9, IL-15, and IL-21 use this receptor chain (44). According to the research, JAK3 and the cytokines IL-4 have a significant role in the etiology of vitiligo. They may be the focus of a promising new treatment for vitiligo that is resistant to current therapies. The JAK1/JAK3 inhibitor tofacitinib abrogates IL-4 signaling and the differentiation of Th2 cells (45); moreover, tofacitinib is an effective and well-tolerated therapy option for people with refractory vitiligo, according to numerous research (46–49). However, the cytokine microenvironment in vitiligo appears to be complicated, as patients exhibit various cytokine signatures at various stages. Our experimental results are not quite consistent with some results. Nouri-Koupae’s study revealed that in patients with vitiligo compared with controls, mRNA expression was significantly higher for IFN-γ and significantly lower for IL-4 mRNAs (13). Parallel to that, Zhen et al. (50) illustrated that in vitiligo patients, the Th17 reaction is much greater than the normal Th1 response by using IL-4 levels in blood samples as a Th2 indicator and IL-17 as a Th17 indicator. The illness Th1(TNF-α, IFN-γ) is traditionally assumed to predominate in vitiligo. The research also discovered vitiligo following the TNF-α inhibitor infliximab injection, which may be difficult to explain based on the abovementioned idea (51–53). Meanwhile, there is mounting evidence that the immunological milieu of vitiligo is also characterized by cytokines connected to other Th2 cell responses, such as IL-4, IL-5, IL-10, IL-13, and IL-31, rather than just Th1 cells and related cytokines (IFN-γ, TNF-α, and IL-2) (25, 35, 54). Hence, tofacitinib, a JAK3 inhibitor designed to lower IL-4-related Th2 cytokines, may thus be useful in treating certain cases of refractory vitiligo. Intriguingly, Nihei et al. (55) used IL-4 inhibitors to treat vitiligo patients; most patients had effective control of their cutaneous lesions, and no overtly negative side effects were discovered. This shows the reasonability and scientificity of IL-4 inhibition in treating some vitiligo patients. There might be several reasons for these conflicting results. For instance, the cytokine pattern in vitiligo patients may vary greatly depending on the severity of the illness and the degree of body region involvement. Moreover, due to the involvement of outside variables (drugs and environment), immunological skin disorders often characterized by Th1-type immune responses can occasionally change to Th2-type immune responses, leading to medication resistance and making treatment challenging (56). The significance of this study is to recognize the diversity of immune responses in vitiligo, not only to stay limited to Th1-type responses. We must admit that the type of immune response in vitiligo is not static but can change with the severity of the disease, progression, and location, leading to the traditional treatment effect becoming particularly difficult and drug resistance. When conventional treatments are ineffective, patients with vitiligo can receive therapies such as JAK1/3 inhibitors or an IL-4 monoclonal antibody inhibitor to manage their condition.

Our MR analysis provides more trustworthy data for determining the causal association between circulating cytokine concentrations and the chance of developing vitiligo compared to typical observational studies because it overcomes the bias brought on by confounding and reverse causality problems. Our MR investigation has a few constraints, which could affect the conclusion of the relationship between cytokines and vitiligo. First, the cytokine GWAS data set was relatively small, which reduced the statistical power of causal assessment in MR. It might illustrate why biologics used in managing vitiligo, such as IFN-γ, IL-1, and IL-2, which played a critical role in vitiligo, did not provide statistically significant effects in our MR analysis. Secondly, most individuals in the two GWASs were of European heritage, so further research is needed to determine whether our findings are applicable. Next, the present cytokine GWAS research methodologies constrain the depth of our investigation. The generalizability of our results and our study’s precision may be enhanced by adopting an advanced analytical approach to boost the specificity and accuracy of the existing results. More significantly, given the inherent limits of MR studies, it would be desirable to investigate the connection between blood levels of IL-4 and the severity of vitiligo in a large group of vitiligo patients in the future to validate the previous findings. By merging the information from cohort studies, clinical trials, and functional investigations, further studies must be conducted to find connections between cytokines and vitiligo. This inquiry helps examine the pathophysiology of vitiligo.

## 5 Conclusion

In the final analysis, our bidirectional MR study demonstrated evidence of a possible causal relationship between vitiligo and certain cytokine levels. Our results would provide further evidence in favor of cytokine-targeted treatment for vitiligo and create a solid foundation for future investigations into the pathophysiology of the cytokine responsible for the condition.

## Data availability statement

Publicly available datasets were analyzed in this study. This data can be found at: (https://thl.fi/en/web/thlfi-en/research-and-expertwork/population-studies/the-national-finrisk-study), https://gwas.mrcieu.ac.uk/datasets/finn-b-L12_VITILIGO/.

## Ethics statement

No new information was acquired, and no new ethical analysis was conducted. The studies were conducted in accordance with the local legislation and institutional requirements. Written informed consent for participation was not required from the participants or the participants' legal guardians/next of kin in accordance with the national legislation and institutional requirements.

## Author contributions

CL: Conceptualization, Data curation, Formal analysis, Funding acquisition, Investigation, Methodology, Software, Writing – original draft, Writing – review & editing. XLiu: Data curation, Investigation, Methodology, Supervision, Writing – original draft, Writing – review & editing. HX: Funding acquisition, Resources, Supervision, Validation, Visualization, Writing – original draft, Writing – review & editing. XLi: Funding acquisition, Resources, Writing – original draft, Writing – review & editing.
